# Quantitative and semi-quantitative assessment of synovitis on MRI and the relationship with symptoms in symptomatic knee osteoarthritis

**DOI:** 10.1093/rheumatology/keaa619

**Published:** 2020-10-23

**Authors:** Thomas A Perry, Xiaotian Yang, James van Santen, Nigel K Arden, Stefan Kluzek

**Affiliations:** 1 Nuffield Department of Orthopaedics, Rheumatology and Musculoskeletal Sciences, Botnar Research Centre, Oxford, UK; 2 Versus Arthritis Centre for Sport, Exercise and Osteoarthritis, University of Oxford, Oxford, UK; 3 Department of Rehabilitation Medicine, Sir Run Run Shaw Hospital, Zhejiang University School of Medicine, Hangzhou, China; 4 MRC Lifecourse Epidemiology Unit, University of Southampton, Southampton General Hospital, Southampton, UK; 5 Division of Rheumatology, Orthopaedics and Dermatology, School of Medicine, University of Nottingham, Nottingham, UK

**Keywords:** synovitis, knee, osteoarthritis, MRI, quantitative, semi-quantitative

## Abstract

**Objectives:**

Synovitis in symptomatic knee OA (KOA) is common and is associated with joint symptoms. Optimal synovial measurement on MRI is, however, unclear. Our aims were to examine the relationship between MRI measures of synovitis and knee symptoms in symptomatic KOA.

**Methods:**

Data from a randomized, multicentre, placebo-controlled trial (UK-VIDEO) of vitamin-D therapy in symptomatic KOA were utilized. Participants reported knee symptoms using WOMAC at baseline and annually. On contrast-enhanced (CE) MRI, synovial thickness was measured using established, semi-quantitative methods whilst synovial tissue volume (STV) was assessed as absolute STV (aSTV) and relative to the width of femoral condyle (rSTV). STV of the infrapatellar region was also assessed. Associations between synovial measures and symptoms were analysed using multiple linear regression modelling.

**Results:**

No linear association was observed between knee symptoms and synovitis thickness scores. Whole-joint aSTV (0.88, 95% CI: 0.17, 1.59) and infrapatellar aSTV (5.96, 95% CI: 1.22, 10.7) were positively associated with knee pain. Whole-joint rSTV had a stronger association with pain (7.96, 95% CI: 2.60, 13.33) and total scores (5.63, 95% CI: 0.32, 10.94). Even stronger associations were found for infrapatellar rSTV with pain (55.47, 95% CI: 19.99, 90.96), function (38.59, 95% CI: 2.1, 75.07) and total scores (41.64, 95% CI: 6.56, 76.72).

**Conclusions:**

Whole-joint and site-specific infrapatellar STV measures on CE-MRI were associated with knee pain, respectively. Volumes relative to the size of the femoral condyle may be promising outcome measures in KOA trials.

**Rheumatology key messages**

- Synovial tissue volume (STV) measures were strongly, and linearly, associated with knee symptoms in symptomatic KOA.
- Whole-joint absolute STV and site-specific absolute infrapatellar STV were positively associated with knee pain.
- STV measures relative to the size of the femur were strongly associated with knee symptoms.

## Introduction

OA of the knee is the most common cause of knee pain among those aged 45 years and older [1] and there is evidence to suggest that prevalence is increasing [2, 3]. Synovial inflammation is present at all stages of knee OA (KOA) and is most likely a secondary phenomenon. Currently, there are no licensed disease-modifying treatments for knee OA with currently available interventions aimed at alleviating painful symptoms and improving function. The current focus is to identify clinically important tissue targets that are related to the underlying pathogenesis of OA.

Synovitis has an important role in KOA [4] with data suggesting that synovitis is an early risk factor for the development of radiographic KOA [5–7] and rapid-onset ‘accelerated KOA’ [8]. Further, data from most observational studies, but not all, suggest that synovitis is associated with structural progression with the evidence suggesting that only those with severe synovitis are at greater risk of progressing in their disease [7, 9, 10]. There is also evidence to suggest that synovitis is associated with pain [11–14], with the degree of synovitis correlating with the degree of knee pain in a dose-response manner [15]. OA is widely accepted to be a heterogeneous disease [16] thus it is likely that the severity of synovitis is likely to fluctuate with symptoms. Synovitis has been shown to resolve in a relatively short time [11] and so has become a prominent treatment target in KOA trials. Currently, there is no consensus over the optimum approach for the assessment of synovitis on MRI in KOA.

There are several markers of synovitis on MRI, which include the volume of joint effusion and increased signal of thickened synovium on fluid sensitive sequences and, the volume of contrast-enhanced (CE) synovial membrane [17–20]. In large epidemiological imaging studies, non-CE MRI is frequently used [10, 21, 22] though this technique does not allow for optimum differentiation of effusion from synovium [23]. Subsequently, CE-MRI is the preferred method of assessment; with data from histological studies supporting strong correlations between CE-MRI and macroscopic evaluation [23]. Furthermore, signal changes in Hoffa’s fat pad (infrapatellar region) have been used as surrogate markers of synovial activation [24]; a sensitive yet non-specific maker of synovitis when using CE-MRI as the reference standard [25]. Synovitis in KOA is frequently assessed using semi-quantitative methods [22, 26, 27], in which scores are used to grade severity of synovitis distension, and quantitatively in which the volume of the synovial tissue is directly measured [28].

Imaging biomarkers used to evaluate synovitis are likely to play an increasing role in identifying participants for OA clinical trials and assessing treatment efficacy in which synovitis is the treatment target. Our aim was to explore the relationship between different measures of synovitis and symptoms in symptomatic KOA using CE-MRI.

## Methods

### Data source and sample size

We used cross-sectional data from the Vitamin D in OA (UK VIDEO) trial [29] (ISRCTN94818153). VIDEO was a 3-year double-blind, randomized, placebo-controlled trial involving five UK NHS centres, which examined the effect of vitamin D therapy on radiographic joint space narrowing and symptoms in men and women with symptomatic KOA [29]. Details of the original VIDEO study have been published previously [29]. In brief, eligible participants had radiological evidence of KOA at the medial tibio-femoral knee compartment [Modified Kellgren & Lawrence (K&L) [30] score of 2–3], joint space width >1 mm and had knee pain for most days of the previous month. A total of 474 participants were randomized, within centres, to receive either oral vitamin D (800 IU/daily) or matching placebo in a 1:1 ratio.

A subsample of participants recruited at Southampton (*n* = 174) had MRIs performed. Participants eligible for this study had sagittal and/or axial T_1_-weighted (T_1_-w) fat suppressed (FS) CE MRIs, an axial proton-density-weighted (PD-w) FS and/or coronal short tau inversion recovery (STIR) scan for the corresponding visit, synovitis thickness scores, synovial tissue volume (STV) measures and WOMAC symptom data at, at least, a single visit across follow-up. Each participant contributed a single visit to the analysis. Ethical approval was not required for any aspect of the work presented in this manuscript.

### Magnetic resonance imaging

Images were acquired on a 1.5 T MRI scanner (Signa (GE Healthcare)) using a dedicated phased-array knee coil [14]. Sagittal and axial post-contrast T_1_-w fat suppressed (FS) (repetition time (TR) = 600–800 ms, echo time (TE) = 12.5–16.2 ms, acquisition matrix 256 × 160, slice gap = 0.6 mm, slice thickness = 3 mm), axial proton density weighted (PD-w) FS (TR = 3800–4820 ms, TE = 31.2–32.5 ms, matrix 256 × 192, slice gap = 0.2 mm, slice thickness = 4 mm) and coronal short tau inversion recovery (STIR) (TR = 3000–4760 ms, TE = 46.1–56.9 ms, matrix 256 × 192, slice gap = 0.3 mm, slice thickness = 3 mm) sequences were acquired. For image acquisition, participants were positioned supine for scanning. An intravenous injection of gadodiamide [0.2 ml/kg body weight (Omniscan, GE Healthcare)] was administered 3 minutes prior to the acquisition of the first CE scan [14]; with all scans acquired within 11 minutes of contrast administration.

### Quantitative assessment of synovial tissue volume

A single reader (T.A.P) performed segmentation of STV (mm^3^) using a semi-automated approach that has been described previously [31]. In brief, the software segments enhancing STV (on CE-MRI) based on a threshold, as selected by the user, within a 3 D-mask that is applied to the target image [31]. STV measured across the whole joint was termed total absolute STV (aSTV). Segmentation of STV was performed using sagittal T_1_-w fat suppressed CE scans on the index (most painful) knee. Reliability of STV assessment has been assessed previously and was found to be excellent for both intra- (ICC_3,1_=0.99) and inter-observer agreement (ICC_3,1_=0.83) [13].

### Relative measures of STV and infrapatellar synovitis volume

**Fig. 1:**
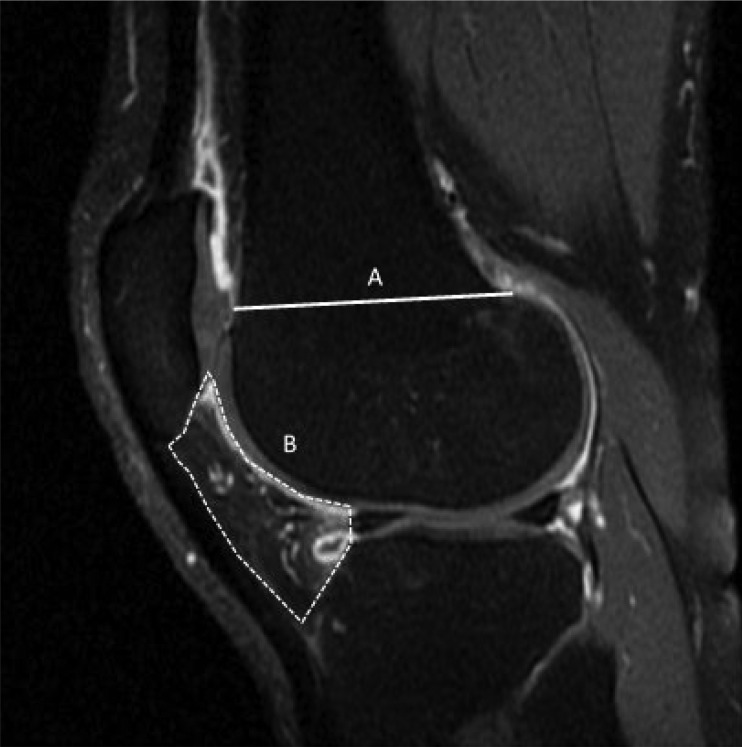
Sagittal T_1_-w fat suppressed (FS) contrast-enhanced MRI of a symptomatic osteoarthritic knee (**A**) Delineation of the femur for femoral condyle width measurement and (**B**) region of interest defined for the infrapatellar (Hoffa’s) fat pad.

The total STV in the joint compartment relative to femoral condyle width was calculated and was expressed as a single outcome; total relative STV (rSTV). To calculate relative volume, we assessed the width of the femoral condyle on a total of four consecutive slices (where the femoral condyle was at its most visible and at its greatest dimension) for each participant. All images were maximized within the software window in order to standardise measurements. Width measurements were manually recorded, on a Dell monitor (1920 × 1080 pixel resolution, 23-inch), for each participant in accordance with criteria defined by Hunter *et al.* [27]; for the anatomical delineation of the femur into trochlea and weight-bearing regions (see Fig. 1). Width measurements were averaged with total relative values calculated as follows; [total absolute STV (mm^3^)/mean femoral width (mm)].

We further segmented high signals on CE-MRI in the infrapatellar region (Fig. 1) and termed this volume total absolute infrapatellar STV; referred throughout as infrapatellar aSTV. The infrapatellar region was defined as the region directly adjacent to the inferior patella pole [27]. We further included high signals directly adjacent to the lateral menisci as there is evidence to suggest that localized meniscal damage is associated with surrounding synovitis [32]. Total relative infrapatellar STV, referred to as infrapatellar rSTV, was calculated as [total absolute infrapatellar STV (mm^3^)/mean femoral width (mm)].

### Semi-quantitative assessment of synovial thickness

Synovial thickness was examined in a subsample (*n* = 107) of VIDEO as part of a previous study [14]. Synovial thickness was assessed using a semi-quantitative grading system which has been previously validated [33]. In brief, a single musculoskeletal radiologist with over 8 years’ experience in image assessment in knee OA scored the MRIs for synovial thickness [14]. An ordinal score of 0–3 [0 = normal, 1 = mild (<2 mm), 2 = moderate (2–4 mm) and 3 = severe (>4 mm)] was assigned across 11 regions including the infrapatellar region. Thickness was assessed on either sagittal or axial post-contrast sequences [14]. We created a composite measure of total synovial thickness that was equal to the sum of all synovitis scores. We then categorized total synovial thickness as normal (0–4), mild (5–8), moderate (9–12) and severe (≥13) in accordance with previous methods [33].

### Assessment of symptoms

Pain, function and stiffness symptoms were assessed using the Western Ontario and McMaster Universities (WOMAC) questionnaire. Each item of WOMAC was scored on a visual analogue scale (VAS) from 0–100 mm (0 = no pain/disability). WOMAC total was generated as a composite of pain, function and stiffness. Scores corresponded to symptoms experienced within the last 48 hrs.

### Statistical methods

Characteristics of the study participants are presented as means and standard deviations for normally distributed variables and medians and inter-quartile range (IQR) for non-normally distributed variables. Data were analysed using STATA (version 15.1, StataCorp., College Station, TX, USA). To examine the relationship between synovitis (continuous or categoric measures) and symptoms, we used multiple linear regression with: (i) total aSTV; (ii) total rSTV; (iii) infrapatellar aSTV; (iv) infrapatellar rSTV; (v) whole joint synovitis thickness score; and (vi) infrapatellar synovitis thickness score as the respective exposures and WOMAC symptoms (pain, function, stiffness and total) as the outcomes. Synovitis score was included as a categoric exposure as it was assumed that the relationship between semi-quantitatively assessed synovitis and knee symptoms would be non-linear. We formally tested the assumptions of linear regression and confirmed that there were no violations. All regression models were adjusted for potential confounders which included age, sex, body mass index (BMI) and the presence/absence of Heberden’s nodes. We did not adjust for the allocation of treatment intervention as it has been previously reported that vitamin D therapy has no observed effect on the outcomes described here [34].

## Results

### Subjects

In total, 96 participants had CE-MRI scans, symptom data, synovitis thickness scores, total aSTV and total rSTV and infrapatellar STV measured at least a single visit across follow-up. Participants’ mean age (s.d.) was 65.4 (± 8.1) years and most participants were female. Table 1 shows the characteristics of the included participants.

**Table 1:** Clinical and imaging characteristics of the study participants (*n* = 96)

Variable	
Females, *n* (%)	63 (65.6)
Index knee, *n* (% Right)	53 (55.2)
Body mass index (BMI) (kg/m^2^)	28.8 (4.6)
Baseline Kellgren–Lawrence grade (medial/lateral) in index knee	
Grade 1, n (%)	19 (19.8)
Grade 2, n (%)	40 (41.6)
Grade 3, n (%)	33 (34.4)
Grade 4, n (%)	4 (4.2)
WOMAC^a^	
Pain score	33.8 (21.0)
Stiffness score	45.8 (25.3)
Function score	37.0 (22.1)
Total score	37.0 (21.1)
Presence of Heberden’s nodes, (yes: *n*, %)	69 (71.9)
Total absolute STV (mm^3^)	9900.6 (6537.7)
Relative total STV	131.8 (86.9)
Total absolute infrapatellar STV (mm^3^)	2012.0 (1042.8)
Total relative infrapatellar STV	26.8 (14.0)

Results are shown as means (s.d.) or frequencies (%). ^a^WOMAC: Western Ontario and McMaster Universities Osteoarthritis Index: visual analogue scale (VAS) used to score pain, function, stiffness and total respectively from 0 to 100 units (0 = no pain / disability, 100 = high pain / disability). STV, synovial tissue volume.

Two participants were excluded from all analysis as they showed evidence of high normalized residual squared values and a high degree of leverage over the regression models (data not shown); exclusion is in line with current recommendations [35]. We assessed a single visit per participant with synovitis measured at the baseline visit in 45 (47.9%) participants, while 22 (23.4%), 23 (24.5%) and 4 (4.2%) participants had 12, 24- and 36-month follow-up visits assessed respectively. WOMAC scores were captured at the time of MRI acquisition in all participants. In those with missing scores (*n* = 2), data at the following visit (6-months post-MRI) was used. All participants had evidence of synovitis on CE-MRI. In addition, whilst inclusion to the primary VIDEO trial specified that all participants must have a KL grade of 2–3, upon re-evaluation of the radiographs some participants were re-graded as KL1s and KL4s.

### Total absolute STV and infrapatellar STV and symptoms

In multivariate analysis, there was a statistically significant association between total aSTV and pain (0.88, 95% CI: 0.17, 1.59), though not with function (0.46, 95% CI: −0.28, 1.19), stiffness (0.53, 95% CI: −0.37, 1.42) and total score (0.55, 95% CI: −0.15, 1.26); see Table 2. Similarly, there was a statistically significant association between infrapatellar aSTV and pain (5.96, 95% CI: 1.22, 10.7); with an increase in volume associated with worsening knee pain (Table 2). There was, however, no statistically significant association between infrapatellar aSTV and function (3.31, 95% CI: −1.54, 8.17), stiffness (2.5, 95% CI: −3.46, 8.46) and total (3.8, 95% CI: −0.88, 8.48).

**Table 2:** Association between synovitis volume and symptoms

Outcome	Univariate (*n* = 94)	Multivariate ^a^ (*n* = 94)	Multivariate^b^ (*n* = 94)	Multivariate^c^ (*n* = 94)
Total absolute STV^d^				
WOMAC^e^ pain	**0.89 (0.17, 1.61), 0.02**	**0.89 (0.17, 1.62), 0.02**	**0.93 (0.22, 1.64), 0.01**	**0.88 (0.17, 1.59), 0.02**
Stiffness	0.48 (−0.42, 1.38), 0.29	0.49 (−0.4, 1.38), 0.28	0.53 (−0.36, 1.41), 0.24	0.53 (−0.37, 1.42), 0.24
Function	0.41 (−0.37, 1.19), 0.3	0.41 (−0.37, 1.19), 0.3	0.49 (−0.24, 1.21), 0.19	0.46 (−0.28, 1.19), 0.22
Total	0.52 (−0.23, 1.26), 0.17	0.52 (−0.22, 1.26), 0.17	0.58 (−0.11, 1.28), 0.1	0.55 (−0.15, 1.26), 0.12
Total absolute infrapatellar STV^d^				
Pain	**5.4 (0.63, 10.16), 0.03**	**5.86 (1.07, 10.65), 0.02**	**6.31 (1.6, 11.03), 0.01**	**5.96 (1.22, 10.7), 0.01**
Stiffness	1.41 (−4.51, 7.34), 0.64	2.15 (−3.76, 8.06), 0.47	2.5 (−3.38, 8.38), 0.4	2.5 (−3.46, 8.46), 0.41
Function	2.09 (−3.04, 7.22), 0.42	2.63 (−2.52, 7.78), 0.31	3.5 (−1.3, 8.31), 0.15	3.31 (−1.54, 8.17), 0.18
Total	2.73 (−2.16, 7.62), 0.27	3.27 (−1.63, 8.16), 0.19	4.01 (−0.62, 8.64), 0.09	3.8 (−0.88, 8.48), 0.11

All results presented with 95% CIs and *P*-values. Statistically significant results (*P* ≥0.05) are shown in bold. ^a^Adjusted for sex. ^b^Adjusted for sex, age, body mass index (BMI). ^c^Adjusted for sex, age, body mass index (BMI) and presence of Heberden’s nodes. ^d^Absolute effects sizes from the linear regression models reflect the change in WOMAC score for a 1 cm^3^ increase in volume. ^e^WOMAC = Western Ontario and McMaster Universities Osteoarthritis Index: visual analogue scale (VAS) used to score knee symptoms from 0–100 (0 = no pain/disability to 100 = high pain/disability). STV, synovial tissue volume.

### Total relative STV and infrapatellar STV and symptoms

In multivariate analysis, there was a statistically significant association between total rSTV and knee pain (7.96, 95% CI: 2.60, 13.33) and total score (5.63, 95% CI: 0.32, 10.94) though there was no association with function (4.96, 95% CI: −0.56, 10.49) and stiffness (5.47, 95% CI: −1.29, 12.22), respectively; see Table 3. Infrapatellar rSTV was statistically significantly associated with pain (55.47, 95% CI: 19.99, 90.96), function (38.59, 95% CI: 2.1, 75.07) and total score (41.64, 95% CI: 6.56, 76.72) though not with stiffness (32.61, 95% CI: −12.45, 77.67). The reported coefficients for these infrapatellar rSTVs appear large (e.g. the coefficient of 55.47 implies that for a 1 unit increase in infrapatellar rSTV, this reflects a corresponding change in WOMAC pain score of 55.47 points), though this accurately reflects volumes that are typically small.

**Table 3:** Association between relative synovitis volume and symptoms

Outcome	Univariate (*n* = 94)	Multivariate^a^ (*n* = 94)	Multivariate^b^ (*n* = 94)	Multivariate^c^ (*n* = 94)
Total relative STV^d^				
WOMAC^e^ pain	**8.15 (2.7, 13.6), 0.004**	**8.02 (2.56, 13.48), 0.004**	**8.29 (2.93, 13.64), 0.003**	**7.96 (2.60, 13.33), 0.004**
Stiffness	5.47 (−1.34, 12.29), 0.11	5.19 (−1.58, 11.95), 0.13	5.45 (−1.24, 12.13), 0.11	5.47 (−1.29, 12.22), 0.11
Function	4.84 (−1.08, 10.75), 0.11	4.64 (−1.27, 10.54), 0.12	5.13 (−0.35, 10.61), 0.07	4.96 (−0.56, 10.49), 0.08
Total	5.58 (−0.03, 11.19), 0.051	5.39 (−0.21, 10.99), 0.06	**5.82 (0.55, 11.09), 0.03**	**5.63 (0.32, 10.94), 0.04**
Total relative infrapatellar STV^d^				
Pain	**52.92 (16.78, 89.06), 0.005**	**54.26 (18.17, 90.35), 0.004**	**57.33 (21.84, 92.81), 0.002**	**55.47 (19.99, 90.96), 0.003**
Stiffness	27.9 (−17.5, 73.3), 0.23	30.29 (−14.65, 75.23), 0.18	32.59 (−12.07, 77.24), 0.15	32.61 (−12.45, 77.67), 0.15
Function	31.75 (−7.43, 70.93), 0.11	33.46 (−5.54, 72.46), 0.09	**39.53 (3.29, 75.76), 0.03**	**38.59 (2.1, 75.07), 0.04**
Total	35.87 (−1.33, 73.08), 0.06	**37.56 (0.59, 74.56), 0.047**	**42.69 (7.82, 77.56), 0.02**	**41.64 (6.56, 76.72), 0.02**

All results presented with 95% CIs and *P*-values. Statistically significant results (*P* ≥0.05) are shown in bold. ^a^Adjusted for sex. ^b^Adjusted for sex, age, body mass index (BMI). ^c^Adjusted for sex, age, body mass index (BMI) and presence of Heberden’s nodes. ^d^Absolute effects sizes from the linear regression models reflect the change in WOMAC score for a 1 cm^3^ increase in relative volume. ^e^WOMAC = Western Ontario and McMaster Universities Osteoarthritis Index: visual analogue scale (VAS) used to score knee symptoms from 0–100 (0 = no pain/disability to 100 = high pain/disability). STV, synovial tissue volume.

### Total synovitis thickness scores and symptoms

In multivariate analysis, there was no statistically significant association between severity of whole joint synovitis thickness scores and symptoms (see Table 4). In participants with mild synovial thickness, there was no statistically significant association with pain (4.71, 95% CI: −12.29, 21.7), stiffness (−1.55, 95% CI: −22.48, 19.38), function (5.64, 95% CI: −11.59, 22.86) and total score (4.86, 95% CI: −11.77, 21.5). Further, there was no statistically significant association between moderate synovial thickness and pain (0.46, 95% CI: −16.14, 17.06), stiffness (4.86, 95 CI%: −15.58, 25.3), function (6.73, 95% CI: −10.09, 23.54) and total score (5.29, 95% CI: −10.96, 21.53). Lastly, there was no statistically significant association between severe synovial thickness and pain (9.22, 95% CI: −5.97, 24.41), stiffness (7.04, 95% CI: −11.67, 25.75), function (8.04, 95% CI: −7.35, 23.43) and total score (8.22, 95% CI: −6.65, 23.09).

**Table 4:** Association between total synovitis thickness severity and symptoms

Outcome	Univariate (*n* = 94)	Multivariate^a^ (*n* = 94)	Multivariate^b^ (*n* = 94)	Multivariate^c^ (*n* = 94)
Total synovitis thickness score^d^				
**WOMAC** ^e^ **pain**				
Mild (*n*=16)	3.86 (−13.45, 21.17), 0.66	3.58 (−13.77, 20.93), 0.68	3.87 (−13.28, 21.02), 0.66	4.71 (−12.29, 21.7), 0.58
Moderate (*n*=20)	0.07 (−16.61, 16.75), 0.99	−0.11 (−16.81, 16.6), 0.99	0.09 (−16.68, 16.86), 0.99	0.46 (−16.14, 17.06), 0.96
Severe (*n*=49)	7.16 (−7.91, 22.23), 0.35	6.53 (−8.63, 21.69), 0.39	7.8 (−7.46, 23.07), 0.31	9.22 (−5.97, 24.41), 0.23
**Stiffness**				
Mild	−1.89 (−22.96, 19.17), 0.86	−2.51 (−23.41, 18.39), 0.81	−1.68 (−22.46, 19.1), 0.87	−1.55 (−22.48, 19.38), 0.88
Moderate	4.1 (−16.19, 24.39), 0.69	3.7 (−16.42, 23.83), 0.72	4.81 (−15.51, 25.12), 0.64	4.86 (−15.58, 25.3), 0.64
Severe	6.05 (−12.29, 24.38), 0.51	4.64 (−13.62, 22.9), 0.62	6.82 (−11.67, 25.32), 0.47	7.04 (−11.67, 25.75), 0.46
**Function**				
Mild	6.03 (−12.3, 24.35), 0.52	5.59 (−12.68, 23.87), 0.55	5.2 (−11.97, 22.37), 0.55	5.64 (−11.59, 22.86), 0.52
Moderate	7.96 (−9.69, 25.62), 0.37	7.69 (−9.91, 25.28), 0.39	6.53 (−10.26, 23.32), 0.44	6.73 (−10.09, 23.54), 0.43
Severe	7.55 (−8.4, 23.5), 0.35	6.56 (−9.41, 22.52), 0.42	7.3 (−7.99, 22.58), 0.35	8.04 (−7.35, 23.43), 0.3
**Total**				
Mild	4.93 (−12.57, 22.44), 0.58	4.52 (−12.93, 21.97), 0.61	4.37 (−12.25, 20.98), 0.6	4.86 (−11.77, 21.5), 0.56
Moderate	6.02 (−10.84, 22.88), 0.48	5.75 (−11.05, 22.56), 0.5	5.07 (−11.18, 21.31), 0.54	5.29 (−10.96, 21.53), 0.52
Severe	7.36 (−7.87, 22.59), 0.34	6.41 (−8.84, 21.66), 0.41	7.38 (−7.41, 22.16), 0.32	8.22 (−6.65, 23.09), 0.28

All results were in comparison to normal (0 = score) levels of synovial thickness (*n* = 9). All results presented with 95% CIs and *P*-values. ^a^Adjusted for sex. ^b^Adjusted for sex, age, body mass index (BMI). ^c^Adjusted for sex, age, body mass index (BMI) and presence of Heberden’s nodes. ^d^Absolute effects sizes from the linear regression models refer to the increase in WOMAC score for a 1 unit increase in severity score. ^e^WOMAC = Western Ontario and McMaster Universities Osteoarthritis Index: visual analogue scale (VAS) used to score knee symptoms from 0–100 (0 = no pain/disability to 100 = high pain/disability).

### Infrapatellar synovitis thickness scores and symptoms

In a fully adjusted model, there was no statistically significant association between mild infrapatellar synovitis thickness and pain (2.56, 95% CI: −7.8, 12.91), stiffness (−1.39, 95% CI: −14.12, 11.34), function (3.37, 95 CI: −6.95, 13.7) and total score (2.81, 95% CI: −7.21, 12.83); see Table 5. Further, there was no statistically significant association between moderate infrapatellar synovitis thickness and pain (−4.00, 95% CI: −14.96, 6.97), stiffness (−0.43, 95% CI: −13.91, 13.05), function (−5.55, 95% CI: −16.49, 5.39) and total score (−4.8, 95% CI: −15.41, 5.82). Lastly, there was no statistically significant association between severe infrapatellar synovitis thickness and pain (10.09, 95% CI: −15.89, 36.07), stiffness (10.58, 95% CI: −21.35, 42.52), function (−0.76, 95% CI: −26.67, 25.15) and total score (2.45, 95% CI: −22.69, 27.59).

**Table 5:** Association between infrapatellar synovitis thickness severity and symptoms

Outcome	Univariate (*n* = 94)	Multivariate^a^ (*n* = 94)	Multivariate^b^ (*n* = 94)	Multivariate^c^ (*n* = 94)
Total infrapatellar synovitis thickness score^d^				
**WOMAC** ^e^ **pain**				
Mild (*n*=33)	2.79 (−7.32, 12.89), 0.59	2.31 (−7.84, 12.45), 0.65	1.63 (−8.73, 12.0), 0.76	2.56 (−7.8, 12.91), 0.63
Moderate (*n*=23)	−3.57 (−14.75, 7.61), 0.53	−3.69 (−14.86, 7.49), 0.51	−3.88 (−14.93, 7.17), 0.49	−4.00 (−14.96, 6.97), 0.47
Severe (*n*=3)	7.84 (−17.22, 32.9), 0.54	9.28 (−15.92, 34.47), 0.47	10.76 (−15.41, 36.92), 0.42	10.09 (−15.89, 36.07), 0.44
**Stiffness**				
Mild	0.1 (−12.25, 12.45), 0.99	−0.87 (−13.13, 11.39), 0.89	−1.42 (−13.99, 11.15), 0.82	−1.39 (−14.12, 11.34), 0.83
Moderate	−0.04 (−13.7, 13.63), 0.99	−0.27 (−13.78, 13.24), 0.97	−0.42 (−13.82, 12.98), 0.95	−0.43 (−13.91, 13.05), 0.95
Severe	5.38 (−25.24, 36.01), 0.73	8.28 (−22.18, 38.73), 0.59	10.6 (−21.13, 42.33), 0.51	10.58 (−21.35, 42.52), 0.51
**Function**				
Mild	6.06 (−4.47, 16.6), 0.26	5.47 (−5.08, 16.02), 0.31	2.79 (−7.46, 13.05), 0.59	3.37 (−6.95, 13.7), 0.52
Moderate	−4.52 (−16.18, 7.14), 0.44	−4.66 (−16.29, 6.96), 0.43	−5.48 (−16.41, 5.45), 0.32	−5.55 (−16.49, 5.39), 0.32
Severe	0.25 (−25.88, 26.38), 0.99	2.02 ( −24.18, 28.22), 0.88	−0.34 ( −26.22, 25.54), 0.98	−0.76 (−26.67, 25.15), 0.95
**Total**				
Mild	4.89 (−5.22, 15.0), 0.34	4.29 (−5.82, 14.4), 0.4	2.21 (−7.75, 12.16), 0.66	2.81 (−7.21, 12.83), 0.58
Moderate	−3.94 (−15.13, 7.24), 0.49	−4.09 (−15.23, 7.05), 0.47	−4.72 (−15.34, 5.89), 0.38	−4.8 (−15.41, 5.82), 0.37
*** ***Severe	2.27 (−22.79, 27.32), 0.86	4.06 (−21.05, 29.17), 0.75	2.89 (−22.25, 28.02), 0.82	2.45 (−22.69, 27.59), 0.85

All results were in comparison to normal (0 = score) levels of synovial thickness (*N* = 35). All results presented with 95% CIs and *P*-values. ^a^Adjusted for sex. ^b^Adjusted for sex, age, body mass index (BMI). ^c^Adjusted for sex, age, body mass index (BMI) and presence of Heberden’s nodes. ^d^Absolute effects sizes from the linear regression models refer to the increase in WOMAC score for a 1 unit increase in severity score. ^e^WOMAC = Western Ontario and McMaster Universities Osteoarthritis Index: visual analogue scale (VAS) used to score knee symptoms from 0–100 (0 = no pain/disability to 100 = high pain/disability).

## Discussion

In this study, using CE-MRI data from a randomized trial of symptomatic KOA, both absolute and relative measures of whole joint STV and site-specific infrapatellar STV were associated with knee symptoms. There was no observed association between synovial thickness scores and knee symptoms.

Several studies have shown an association between semi-quantitative and quantitative MRI measures of synovitis and symptoms in symptomatic KOA though the strength of the relationship has been shown to vary. O’Neill and colleagues reported in a trial of intra-articular corticosteroid injection therapy in symptomatic KOA that synovitis volume was positively associated with increasing knee pain (−1.13, 95% CI: −1.87, −0.39); with a reduction in score indicating an increase in pain [11]. Similarly, in two separate studies using different samples of the UK VIDEO study, synovial thickness scores (1.82, 95% CI: 0.05, 3.58) [14] and whole joint STV (b = 2.2, 95% CI: 0.6, 3.7) [13] were associated with knee pain; with an increase in severity associated with worsening pain. We go beyond these studies to describe the association between different measures of synovitis and symptoms using CE-MRI in the same study sample, thereby allowing for within-study comparisons.

We observed a linear association between STV (absolute and relative measures) and knee pain; however, we did not observe an association between synovitis thickness (whole joint and infrapatellar) scores and symptoms. A possible explanation for this could be due to differences in the structure assessed across CE-MRI sequences. Semi-quantitative scores assess synovial thickness which, whilst a surrogate of synovitis [23, 36], may be inclusive of non-active inflammation with active inflammation. In contrast, quantitative assessment of volume captures active synovial inflammation, as distinct from effusion, represented by signal enhancement at the synovial membrane. Our study may also have been insufficiently powered across levels of synovial thickening to detect true associations. The observed associations between both absolute and relative measures of STV and multiple WOMAC subunits would, however, give confidence that our study was suitably powered.

To our knowledge, this is the first study to have examined the cross-sectional relationship between relative measures of STV and symptoms using CE-MRI in symptomatic KOA. There is some evidence to suggest that the size of the supra-patellar pouch is associated with knee size [37]; thus, we hypothesized that those with a greater bone size may have increased STV independent of disease severity. We observed that whole-joint STV and infrapatellar STV relative to the size of the femoral condyle were more strongly associated with knee pain compared with absolute measures. The method presented here for calculating relative STV and site-specific relative infrapatellar STV could be readily applied to similar studies of symptomatic knee OA. Future work includes applying this method of calculating relative STV measures to other knee OA cohorts and clinical trial datasets; to confirm the suitability and measurement error of this approach.

Evidence from observational studies have shown a relationship between Hoffa’s synovitis and risk of radiographic KOA [5, 6, 38, 39]. There is, however, little data concerning the relationship between Hoffa’s synovitis and pain despite evidence supporting the frequent involvement of the infrapatellar region [24]. Hill *et al.* showed that change in infrapatellar synovitis was associated with change in knee pain (4.89, 95% CI: 0.42, 9.36) [10]. Similarly, we observed a relationship between pain and infrapatellar aSTV (5.96, 95% CI: 1.22, 10.7) and we also observed a relationship between infrapatellar rSTV and pain (55.47, 95% CI: 19.99, 90.96). The magnitude of the effect was, however, greatest when using relative measures, suggesting that there needs to be consideration of the heterogeneity in knee joint size in studies of KOA and synovitis.

The main strength to this study was that the assessment of synovitis was performed on CE-MRI. Although we were unable to compare histological findings against MRI measures of synovitis, there is strong evidence to support the correlations between CE-MRI assessed synovitis and histology [23].

There are several potential limitations to our study. Firstly, this was a secondary analysis of a randomized trial and so the study sample reported here was relatively small (*n* = 94), thereby reducing statistical power and, therefore, our ability to detect true associations. We did, however, observe consistent statistically significant associations between STV and several components of WOMAC across multivariate models, giving confidence to our findings. Further, we measured the femoral condyle width across a total of four slices. While, ideally, femoral condyle width would be measured across all available slices, this was not pragmatic and our methods are in agreement with previous methods used to quantify relative values [40, 41]. Further, we measured femoral condyle width from two points of the subchondral bone, which is in keeping with previous methods [27]. The subchondral bone is, however, subject to OA-related structural changes including erosion and osteophyte development, which may have subsequently affected width measurements; calculating average width measures would, however, reduce the influence of such structures (if present) on the final calculated relative values. A further limitation is the generalisability of our infrapatellar STV measure. There is great variability in the definition of Hoffa’s synovitis [26, 27, 33]. We used a modified version of BLOKS [27] to define infrapatellar STV, which included synovitis directly adjacent to the fat pad and within the infrapatellar region and the intercondylar region extending posteriorly to the lateral menisci. Subsequently, we were unable to differentiate origins of inflammation; that is, inflammation having occurred in response to infrapatellar activation or injury at the menisci. Cartilage volume, meniscal extrusion/injury and bone marrow lesion (BML) volume were not captured as part of the original protocol and so were not adjusted for in our analysis. Subsequently, while we observed a strong, statistically significant relationship between measures of synovitis and knee symptoms, we cannot completely exclude the contribution of the other structural features to knee symptoms. Further work is required to confirm these findings. Lastly, due to the cross-sectional design of the study, we were unable to determine causality. While cross-sectional, the precision of our estimates were, however, likely improved with the addition of data across multiple study visits.

In conclusion, we observed an association between absolute measures of whole-joint STV and site-specific infrapatellar STV and knee symptoms respectively in symptomatic knee OA. Relative measures were more strongly associated with knee symptoms and there was no linear association between synovial thickness and knee symptoms. Relative measures of STV may prove useful outcomes in trials of knee OA.

## Data Availability

All data generated and analysed in this study are available upon reasonable request. Access to data generated in this report should be sent to the corresponding author at thomas.perry@ndorms.ox.ac.uk.
